# In silico and gene expression analysis of the acute inflammatory response of gilthead seabream (*Sparus aurata*) after subcutaneous administration of carrageenin

**DOI:** 10.1007/s10695-021-00999-6

**Published:** 2021-08-26

**Authors:** Jose Carlos Campos-Sánchez, Javier Mayor-Lafuente, Francisco A. Guardiola, María Ángeles Esteban

**Affiliations:** Immunobiology for Aquaculture Group, Department of Cell Biology and Histology, Faculty of Biology, University of Murcia, Campus Regional de Excelencia Internacional “Campus Mare Nostrum”, 30100 Murcia, Spain

**Keywords:** Carrageenin, Skin inflammation, In silico analysis, Gene expression, Teleosts, Aquaculture

## Abstract

Inflammation is one of the main causes of loss of homeostasis at both the systemic and molecular levels. The aim of this study was to investigate in silico the conservation of inflammation-related proteins in the gilthead seabream (*Sparus aurata L.*). Open reading frames of the selected genes were used as input in the STRING database for protein–protein interaction network analysis, comparing them with other teleost protein sequences. Proteins of the large yellow croaker (*Larimichthys crocea L.*) presented the highest percentages of identity with the gilthead seabream protein sequence. The gene expression profile of these proteins was then studied in gilthead seabream specimens subcutaneously injected with carrageenin (1%) or phosphate-buffered saline (control) by analyzing skin samples from the injected zone 12 and 24 h after injection. Gene expression analysis indicated that the mechanisms necessary to terminate the inflammatory response to carrageenin and recover skin homeostasis were activated between 12 and 24 h after injection (at the tested dose). The gene analysis performed in this study could contribute to the identification of the main mechanisms of acute inflammatory response and validate the use of carrageenin as an inflammation model to elucidate these mechanisms in fish.

## Introduction

Inflammation is a temporary local response characterized by heat, redness, swelling, pain, and functional disorders (Nathan 2002). It is triggered by the innate immune system to protect the host against tissue damage, and its aim is to restore physiological functions when homeostatic mechanisms are insufficient (Medzhitov 2008). Inflammation consists of a sequence of events. Depending on diverse factors (such as the causal agent of the inflammation or the anatomical site where it is triggered), inflammatory mediators (including prostaglandins, histamines, cytokines, reactive oxygen species, and enzymes) are released by tissue-resident immune and non-immune cells to increase the blood flow and cause vasodilation (Calder et al. 2013). Capillary permeability is subsequently increased due to the retraction of endothelial cells, allowing access of many large and soluble molecules to the inflammation site (Calder et al. 2013). The third main event is the recruitment of leucocytes (granulocytes, neutrophils, eosinophils and basophils, and monocytes-macrophages) from the blood capillaries to the surrounding tissues and subsequently to the inflammation site due to the repertoire of chemokines and adhesion molecules released by endothelial cells (Calder et al. 2013). Finally, inhibitory mechanisms are normally activated to terminate the inflammatory process and initiate the repair of damaged tissue (Calder et al. 2013), thus preventing unnecessary damage and restoring homeostasis (Chen and Nuñez 2010; Henson 2005). However, if these mechanisms fail to restore homeostasis, inflammation may persist, resulting in a pathological state of chronic inflammation and even chronic diseases (Nathan and Ding 2010; Serhan et al. 2011).

Although inflammatory processes have been extensively studied in mammals, they have not been sufficiently studied in fish. The intensive fish production in aquaculture, one of the fastest-growing sectors in the world in the past few decades, is very frequently associated with inflammation caused injuries, especially wounds and ulcers in the skin of farmed fish (Chen and Nuñez 2010). Moreover, fish are highly susceptible to stress due to the intimate contact with their environment, which can compromise their welfare and promote illness (Delamare-Deboutteville et al. 2006). The final consequences of inflammation and the outbreak of diseases in fish are substantial economic losses (Balcázar et al. 2006; Esteban 2012). Therefore, the search and use of non-invasive methods, such as in silico analyses, can be beneficial for the study of molecular processes that have scarcely been studied in fish, such as inflammation (Liu et al. 2014; Romano and Tatonetti 2019). Exploring the conservation of pro-inflammatory and anti-inflammatory proteins and other inflammation-related molecules among species, as well as their functions, could contribute to the identification of the patterns and signal transduction pathways that regulate this complex process (Hawiger and Zienkiewicz 2019). Since physical interactions between proteins play an important role in this process, study methods are increasingly based on predicting protein–protein interactions purely from protein sequences to identify proteins that have not been previously studied (Makrodimitris et al. 2020). The public online database Search Tool for the Retrieval of Interacting Genes and Proteins (STRING; https://string-db.org/) provides accurate information on protein associations (interactions) by combining different sources and techniques, such as gene co-expression and text mining, and can assign a protein interaction probability score to each physical interaction and functional association (Szklarczyk et al. 2019). These scores have been used to reconstruct protein–protein interaction networks (Kummer et al. 2018). However, the complexity of the studied organisms and the information extracted from the database may vary to such a degree that a protein interaction may not coincide with the organism of interest or may not offer contextual information, necessitating experimental validation of the obtained results (Björne and Salakoski 2019).

In this study, we used carrageenin to better understand the mechanism of inflammation. Carrageenin is a high-molecular-weight sulfated mucopolysaccharide derived from the cell wall of the red algae commonly called Irish moss or carrageen moss (*Chondrus crispus*). It has been used for decades as a model of acute inflammation in rats, mice, and guinea pigs and in in vitro inflammation experiments in humans (Bhattacharyya et al. 2008a, b; Bhattacharyyaa et al. 2010; Levy 1969; Morris 2003; Winter et al. 1962). Carrageenin is composed of 1,3α-1,4β-galactans having one (κ-), two (ι-), or three (λ-) sulfates per disaccharide unit and can produce an inflammatory response associated with edema, hyperalgesia, and erythema and an acute response to thermal and mechanical stimuli. When self-associating into helical structures, κ- and ι-carrageenin can form rigid and flexible gels, respectively, in ionic solutions and seems to be related to the immunostimulant activity against bacterial infections in teleost fish (Cheng et al. 2007; Fujiki et al. 1994, 1997; Necas and Bartosikova 2013). In contrast, λ-carrageenin does not form helices or gels, but it is the main compound responsible for inducing an acute inflammation response when injected in rodents (Fujiki et al. 1997). The few studies investigating the effects of carrageenin on fish have reported that intraperitoneal injection of carrageenin induces acute inflammation characterized by leucocyte infiltration and significant abdominal edema a few hours after administration, peaking several hours later (Huang et al. 2014; Martins et al. 2006; Matushima and Mariano 1996; Ribas et al. 2016). After being injected, carrageenin seems to remain extracellularly for several hours, causing chronic inflammation until macrophages begin to engulf and store it in cytoplasmic vesicles, eventually removing all the extracellular carrageenin by the end of the inflammation process (Timur et al. 1977).

In light of these findings, the aim of this study was to evaluate the involvement of 40 inflammation-related proteins (cellular markers and pro-inflammatory and anti-inflammatory proteins) in the inflammatory process evoked by the subcutaneous administration of carrageenin. Carrageenin was selected because it is known to trigger inflammation in mammals (Levy 1969; Winter et al. 1962; Morris 2003), while the gilthead seabream was chosen as a fish species of interest to marine aquaculture (APROMAR 2019). STRING was used to compare the protein sequence of the genes studied in gilthead seabreams with protein sequences in other similar fish species. The functional associations and interactions between the proteins involved in the same process and their level of conservation were also studied. To our knowledge, this is the first study to evaluate the modulation of gene expression after carrageenin administration in fish.

## Materials and methods

### Protein prediction and functional protein association network analysis

Based on previous inflammation studies on mammals and zebrafish (Campos-Sánchez and Esteban 2021; Chen and Nuñez 2010) and taking into account the published gilthead seabream genome, 40 inflammation-related proteins were selected to investigate the conservation of inflammatory routes. These proteins were divided into three groups: (i) cell marker proteins (colony-stimulating factor 1 receptor [CSF1R], NADPH oxidase subunit Phox22 [PHOX22], NADPH oxidase subunit Phox40 [PHOX40], and major histocompatibility complex class IIa [MHC-II]), (ii) pro-inflammatory proteins (interleukin-1β [IL-1β], tumor necrosis factor alpha [TNF-α], interleukin-6 [IL-6], interleukin-7 [IL-7], interleukin-8 [IL-8], interleukin-18 [IL-18], toll-like receptor 2 [TLR2], toll-like receptor 5 [TLR5], toll-like receptor 7 [TLR7], toll-like receptor 8 [TLR8], toll-like receptor 9 [TLR9], toll-like receptor 13 [TLR13], v-rel avian reticuloendotheliosis viral oncogene homolog A [RelA], v-rel avian reticuloendotheliosis viral oncogene homolog [C-Rel], v-rel avian reticuloendotheliosis viral oncogene homolog B [RelB], nuclear factor of kappa light polypeptide gene enhancer in B-cells 1 [NF-κB1], nuclear factor of kappa light polypeptide gene enhancer in B-cells 2 [NF-κB2], tumor necrosis factor receptor superfamily, member 1a [TNFRSFa], tumor necrosis factor receptor superfamily, member 1b [TNFRSF1b], TNF receptor-associated factor 6 [TRAF6], interleukin-1 receptor-associated kinase 1 [IRAK1], inhibitor of nuclear factor kappa B kinase regulatory subunit gamma [IκBKG], MYD88 innate immune signal transduction adaptor [MYD88], and signal transducer and activator of transcription 3 [STAT3]), and (iii) anti-inflammatory proteins (interleukin-10 [IL-10], transforming growth factor beta 1 [TGF-β1], cathepsin D [CTSD], cathepsin L [CTSL], cathepsin S [CTSS], NLR family CARD domain containing 3 [NLRC3], NLR family, NLR family CARD domain containing 5 [NLRC5 isoforms 1 and 2], NLR family member X1 [NLRX1], acetylcholinesterase [AChE], butyrylcholinesterase [BChE], and cholinergic receptor nicotinic alpha 7 [nAChRα7]).

The gene sequences of the selected proteins were obtained from the gilthead seabream database using the whole-genome shotgun method (Pareek et al. 2011). The open reading frames of such genes were located using the ORFfinder software from the NCBI website (https://www.ncbi.nlm.nih.gov/orffinder/), and an additional check was performed using NCBI Protein BLAST sequence alignment analysis (National Institutes of Health). The protein sequences obtained were used as input in the STRING database for the protein–protein interaction network functional enrichment analysis, comparing sequences between teleosts (superclass Actinopterygii). The percentages of identity of the most similar proteins and the *e*-values were used to investigate the conservation and interactions of gilthead seabream proteins. The protein names were derived automatically based on a cluster’s consensus protein annotations taken from Gene Ontology (GO), the Kyoto Encyclopedia of Genes and Genomes (KEGG), Reactome (2021), UniProt, Pfam, Simple Modular Architecture Research Tool (SMART), and InterPro. The predicted orthologous proteins were associated in networks, where nodes (proteins) and edges (protein–protein associations) denoted the number of predicted interactions between them compared to interactions that would be expected for a random set of proteins of a similar size.

### Design of the primers

The primers used were designed with the Thermo Fisher OligoPerfect™ tool according to the following criteria: (i) each oligonucleotide was composed of 20 nucleotides, (ii) the size of the amplicon had between 100 and 120 nucleotides, (iii) the guanidine-cytosine content was 55–60%, (iv) the semi-naturalization temperature (melting temperature) was as close to 60 °C as possible, and (v) primers that self-inhibit and form hairpins were avoided so as not to hinder the amplification reaction (Table 1).

**Table 1:** Primers used for real-time qPCR

Gene name	Gene abbreviation	GenBank number	Primer sequences (5′ → 3′)	Primer efficiencies
NADPH oxidase, subunit Phox40	*phox40*	AM749961	F: GCGGAGTTGAACCTGAAGAG R: TCACCTTCTGTGTCGCTGTC	106.37%
Colony-stimulating factor receptor 1	*csfr1*	AM050293	F: ACGTCTGGTCCTATGGCATC R: AGTCTGGTTGGGACATCTGG	91.53%
NADPH oxidase, subunit Phox22	*phox22*	FM148169	F: CATCAAGAATCCCCCTCAGA R: TGACAGAGATGGGGTTGTCA	96.26%
Major histocompatibility complex class IIa	*mhcIIa*	DQ019401	F: CTGGACCAAGAACGGAAAGA R: CATCCCAGATCCTGGTCAGT	114.95%
Nuclear factor of kappa light polypeptide gene enhancer in B-cells 2	*nfkb2*	B012900	F: ATCACAGCGCAGAGATCGAG R: TGCGGGATGTAGGTGAACTG	94.67%
Signal transducer and activator of transcription 3	*stat3*	B015325	ACATCCTTGGCACCAACACA ACCATTGCCACACCTCTGTT	97.10%
TNF receptor-associated factor 6	*traf6*	B010645	ACCTGTGTCGTGCCAAGATT TCACAGTACTGGCACGTCAC	96.84%
Toll-like receptor 2	*tlr2*	B008611	F: TCCATGCTTTCGTCCAGGAC R: ACTGTGTTGAGCAAGGCCTC	95.94%
Tumor necrosis factor alpha	*tnf-α*	AJ413189	F: CTGTGGAGGGAAGAATCGAG R: TCCACTCCACCTGGTCTTTC	112.93%
Inhibitor of nuclear factor kappa B kinase regulatory subunit gamma	*ikbkg*	B006470	GAAGGAGGAGGTGGAGCAAC CTCTCTCGCTTCTCGCTCTG	104.24%
MYD88 innate immune signal transduction adaptor	*myd88*	B013233	GCCTTCATCTGCTACTGCCA TCTGTCGAACACGCACAGTT	100.69%
v-rel avian reticuloendotheliosis viral oncogene homolog A	*rela*	B030837	F: GAACCCCACCCTCATGAGTG R: GTTCTGGGCAGCAGTAGAGG	109.93%
Nuclear factor of kappa light polypeptide gene enhancer in B-cells 1	*nfkb1*	B005908	F: CCGACAGACGTTCACAGACA R: TCTTCAGCTGGACGAACACC	98.33%
v-rel avian reticuloendotheliosis viral oncogene homolog	*rel*	B018958	F: AAGCAAGAGCCCCAGATCAC R: TAGGGCGAGGAAGCAAGTTG	104.50%
v-rel avian reticuloendotheliosis viral oncogene homolog B	*relb*	B012502	F: ACAGAGGAGGTGGAGGTCAG R: TATGGATCTGGGTTGTGCGG	106.97%
Tumor necrosis factor receptor superfamily, member 1a	*tnfrsf1a*	B006439	F: TCTTGCGTCTGCTCTCAGTG R: CCTCAGCATCTGGTACTGCC	96.18%
Toll-like receptor 5	*tlr5*	B001824	F: CAACTTGAGCTCCAACGCAC R: GGCTGGAGATAGGTCAAGGC	95.54%
Toll-like receptor 7	*tlr7*	B004477	F: CCAACAATGGGAGCATGGTG R: ATGGTGAGAGTCAGGTTGGTG	104.10%
Interleukin-1 receptor-associated kinase 1	*irak1*	B011814	TGGTGCTGCTGGAGATTCTG AACCGTTCGGACTTTCCTCC	96.39%
Toll-like receptor 8	*tlr8*	B024796	F: CCAGAGCAATTCCAGGGCTA R: TGTCCAGCCCTTTGAACTCTG	90.43%
Interleukin-1	*il-1β*	AJ277166	F: GCGAGCAGAGGCACTTAGTC R: GGTAGGTCGCCATGTTCAGT	103.50%
Toll-like receptor 13	*tlr13*	B003345	F: CCTCCCTGCCTTGACGTATC R: TGTCTGGTTGTTGCTCTGCA	112.59%
Tumor necrosis factor receptor superfamily, member 1b	*tnfrsf1b*	B026296	F: TACCGCAGCTCTTCACGATC R: ACTGTGTGGGGATGCTGATC	98.91%
Interleukin-7	*il-7*	JX976618	F: GATCTGGAAAACACCGGAGA R: TGGACGTGCGTTCTGGTAGC	98.85%
Interleukin-18	*il-18*	JX976626	F: TTGAGGGGTTGTCCTGTTTC R: AGTTTTTACCCCAGCCCTGT	90.32%
Interleukin-6	*il-6*	AM749958	F: AGGCAGGAGTTTGAAGCTGA R: ATGCTGAAGTTGGTGGAAGG	96.80%
Interleukin-8	*il-8*	AM765841	F: GCCACTCTGAAGAGGACAGG R: TTTGGTTGTCTTTGGTCGAA	105.99%
Toll-like receptor 9	*tlr9*	B030920	F: GATCACACCGTTCACTGTCTC R: GGAGGAGAGGGACTGGATTC	98.09%
Acetylcholinesterase	*ache*	B017377	F: CGGAGTGGATGGGTGTGATC R: GTCGGCTCAGTTTCTCCTCC	90.82%
Cathepsin D	*ctsd*	B000122	F: TCGCTGCCTGTTGTCTCTTT R: GCCCGACAGACAGATTGACA	112.76%
Cholinergic receptor, nicotinic, alpha 7	*chrna7*	B000251	F: AATGCCAGCCACAGAGATCC R: TGATTTGGGTCCAGCTCTGC	109.85%
NLR family, CARD domain containing 3	*nlrc3*	B000011	F: CTGCCAGTGGTCAAAGCCTC R: AGGACTGGGAGCTGAGAACT	98.23%
NLR family member X1	*nlrx1*	B002577	F: AGGTGTACCAAAGAGCCACG R: CTGAGGATGGGATGCCAGTC	105.71%
Cathepsin S	*ctss*	B007924	F: AACCTGGTGGACTGTTCGTC R: GCGTCAGAGTCGATACCCTG	106.63%
Cathepsin L	*ctsl*	B019572	F: ATGATGAGCCAGACTGCAGC R: AGACCCCAGCTGTTCTTGAC	113.90%
Butyrylcholinesterase	*bche*	B013682	F: CAGGTACTCCCAACACGGTG R: ATCTCGTAGCCGTGCATGAC	107.75%
Interleukin-10	*il-10*	FG261948	F: CTCACATGCAGTCCATCCAG R: TGTGATGTCAAACGGTTGCT	98.06%
Transforming growth factor 1 beta	*tgf- 1β*	AF424703	F: GCATGTGGCAGAGATGAAGA R: TTCAGCATGATACGGCAGAG	94.54%
NLR family, CARD domain containing 5 (isoform 1)	*nlrc5 (isof.1)*	B003870	F: AGCAGCTAGTTTGGCCTCTG R: GGCGATGTGTTTGATCCCTG	105.93%
NLR family, CARD domain containing 5 (isoform 2)	*nlrc5 (isof.2)*	B003870	F: CAAGAGTGATGCCCCTGTGT R: GACTGTGAGGCTCTGAGCAG	81.50%
Ribosomal protein S18	*rps18*	AM490061	F: CGAAAGCATTTGCCAAGAAT R: AGTTGGCACCGTTTATGGTC	139.43%
Elongation factor-1 alfa	*ef1a*	AF184170	F: TGTCATCAAGGCTGTTGAGC R: GCACACTTCTTGTTGCTGGA	110.84%
Actin beta	*actb*	X89920	F: GGCACCACACCTTCTACAAATG R: GTGGTGGTGAAGCTGTAGCC	103.07%

### Animals

Sixteen specimens (23.7 ± 7 g in weight and 11.7 ± 1 cm in length) of the seawater teleost gilthead seabream, obtained from a local farm (Mazarrón, Spain), were kept in recirculating seawater aquaria (250 L) in the Marine Fish Facilities of the University of Murcia (Spain) for a quarantine period of 1 month. The water temperature was maintained at 20 ± 2 °C with a flow rate of 900 L h^−1^, 28% salinity, and a 12-h light and 12-h dark photoperiod. The water in the tanks was continuously aerated. The fish were fed a commercial diet (Perla MP, Skretting) in quantities amounting to 2% of their body weight daily and were subjected to 24-h fasting before the experiment. Commercial diet consisted of crude protein (48.5%), crude oils and fats (18%), crude ash (6.8%), crude fiber (2.2%), phosphorus (0.9%), calcium (0.9%), and sodium (0.3%). All experimental protocols were conformed to Directive 2010/63/EU and approved by the Ethics Committee of the University of Murcia.

### Experimental design and sample collection

The fish were anesthetized with clove oil (20 mg L^−1^; Guinama), and subcutaneously injected in their left flank, in the middle part between the lateral line and the central zone of the anal fin. Two groups with two replicates (*n* = 4) were randomly established: (i) fish injected with 50 µl of phosphate-buffered saline (PBS; 11.9 mM phosphates, 137 mM NaCl, and 2.7 mM KCl; pH 7.4; Fisher Bioreagents) (control group) and (ii) fish injected with 50 µl of carrageenin in PBS (1%; Sigma-Aldrich) (carrageenin group). Twelve and 24 h after injection, two fish from each tank were randomly caught and sedated as previously described. Skin from the injected area was collected with a biopsy metal punch 4 mm in diameter (Stiefel). The skin samples were immediately stored in TRIzol Reagent (Invitrogen) at − 80 °C for gene expression analysis (Chomczynski 1993).

### Gene expression analysis by real-time qPCR

Total RNA was extracted from 0.5 g samples of gilthead seabream skin using TRIzol, following the manufacturer’s instructions, and quantification and purification were assessed using a Nanodrop® spectrophotometer (the 260:280 ratios were 1.8–2.0). Then, the RNA was treated with DNase I (Promega) to remove genomic DNA contamination and complementary DNA (cDNA) was synthesized from 1 µg of RNA using the reverse transcriptase enzyme SuperScriptIV (Life Technologies) with an oligo-dT_18_ primer. The expression of the nominated genes (see Table 1) was analyzed by real-time qPCR with QuantStudio™ Real-Time PCR System Fast (Life Technologies). The reaction mixtures [containing 5 µl of SYBR Green supermix, 2.5 µl of primers (0.6 µM each), and 2.5 µl of cDNA template] were incubated for 10 min at 95 °C, followed by 40 cycles of 15 s at 95 °C, 1 min at 60 °C, and finally 15 s at 95 °C, 1 min at 60 °C, and 15 s at 95 °C. The gene expression was analyzed using the 2^−ΔCt^ method (Livak and Schmittgen 2001), which was performed as described elsewhere (Cordero et al. 2015). The specificity of the reactions was analyzed using samples without cDNA as negative controls. For each mRNA, gene expression was normalized with the geometric mean of ribosomal protein (*s18*), elongation factor 1-alfa (*ef1α*), and beta-actin (*actb*) RNA content in each sample. Gene names follow the accepted nomenclature for zebrafish (http://zfin.org/). In all cases, each PCR was performed with triplicate samples.

### Statistical analysis

The results were expressed as mean ± standard error of the mean (SEM). Data were analyzed by two-way ANOVA (followed by Tukey tests) to determine differences between experimental groups and each group with respect to time. The normality of the data was previously assessed using a Shapiro–Wilk test and homogeneity of variance was also verified using the Levene test. Non-normally distributed data were log-transformed to perform parametric tests while non-parametric Kruskal–Wallis test, followed by a Dunn’s multiple comparison test, was used when data did not meet parametric assumptions. All statistical analyses were conducted using the computer package SPSS (25.0 version; SPSS Inc., Chicago, IL, USA) for WINDOWS. The level of significance used was *P* < 0.05 for all statistical tests.

## Results

### Protein prediction analysis

To assess the conservation and functional interactions of the proteins involved in the inflammatory process, 40 gilthead seabream proteins were compared with protein sequences of other teleosts that appeared to match the input proteins in the STRING database (Table 2). The large yellow croaker was selected for the analysis due to the higher number of proteins that matched the gilthead seabream protein sequence and the higher homology of its proteins (identity) with those of the gilthead seabream compared to the other teleosts. Gilthead seabream proteins were divided into three groups (cell markers and pro-inflammatory and anti-inflammatory proteins) according to their percentages of identity (Table 3).

**Table 2:** Similar organisms that match the gilthead seabream (*Sparus aurata*) protein sequence ordered according to the number of proteins matched

Organism	Number of proteins matched	Range of proteins identity matched
*Stegastes partitus*	36	93.8–40.2%
*Larimichthys crocea*	35	97.8–44.6%
*Maylandia zebra*	35	96.0–49.3%
*Neolamprologus brichardi*	35	91.7–41.8%
*Oreochromis niloticus*	35	95.9–37.6%
*Oryzias latipes*	35	90.4–32.4%
*Poecilia reticulata*	35	91.2–35.6%
*Xiphophorus maculatus*	35	94.3–35.9%
*Cynoglossus semilaevis*	34	95.5–32.2%
*Haplochromis burtoni*	34	98.7–45.0%
*Poecilia formosa*	34	94.2–39.5%
*Pundamilia nyererei*	34	95.9–46.4%
*Esox lucius*	33	86.6–40.3%
*Gasterosteus aculeatus*	33	94.6–36.8%
*Astyanax mexicanus*	32	93.5–36.1%
*Danio rerio*	31	93.1–36.3%
*Takifugu rubripes*	31	94.6–34.6%
*Lepisosteus oculatus*	30	91.5–32.8%
*Gadus morhua*	28	95.2–38.5%

**Table 3:** Protein prediction analysis between gilthead seabream (*Sparus aurata*) and *Larimichthys crocea* ordered from the highest to the lowest identity

	*S. aurata* proteins	Matching proteins in *L. crocea*	Annotation	Identity	*e*-value
Cell marker	PHOX40	NCF4	Neutrophil cytosol factor 4	91.5%	8.4e-133
	CSF1R	CSF1R1	Macrophage colony-stimulating factor 1 receptor isoform X1	91.0%	0.0
	PHOX22	XP_010739797.1	Cytochrome b-245 light chain	89.7%	8e-85
	MHC-II	XP_010754186.1	The sequence of the model RefSeq protein was modified relative to its source genomic sequence to represent the inferred CDS: added 285 bases not found in genome assembly	67.1%	6.8e-84
Pro-inflammatory proteins	NF-κB2	NFKB2	Nuclear factor NF-kappa-B p100 subunit isoform X1	97.8%	2.4e-205
	STAT3	STAT3	Signal transducer and activator of transcription 3 isoform X1	96.7%	0.0
	TRAF6	TRAF6	TNF receptor-associated factor 6; the sequence of the model RefSeq protein was modified relative to its source genomic sequence to represent the inferred CDS: deleted 1 base in 1 codon	89.1%	6.5e-311
	TLR2	EH28_06854	Toll-like receptor 2 type-2	85.0%	1.7e-108
	TNF-α	EH28_05037	Tumor necrosis factor-like	84.7%	3.5e-28
	IκBKG	IKBKG	Inhibitor of nuclear factor kappa B kinase subunit gamma; NF-kappa-B essential modulator isoform X1	83.2%	2.2e-245
	MYD88	MYD88	Myeloid differentiation primary response protein MyD88	83.0%	1.4e-139
	RelA	RELA	Transcription factor p65; the sequence of the model RefSeq protein was modified relative to its source genomic sequence to represent the inferred CDS: deleted 1 base in 1 codon	82.3%	3.7e-283
	NF-κB1	NFKB1	The sequence of the model RefSeq protein was modified relative to its source genomic sequence to represent the inferred CDS: deleted 4 bases in 3 codons	82.0%	3.8e-36
	C-Rel	EH28_06409	Proto-oncogene c-Rel	81.3%	3e-304
	RelB	RELB	Transcription factor RelB	81.1%	1.5e-265
	TNFRSF1A	EH28_04263	Tumor necrosis factor receptor superfamily member 1A; derived by automated computational analysis using gene prediction method: Gnomon. Supporting evidence includes similarity to: 1 Protein, and 100% coverage of the annotated genomic feature by RNAseq alignments, including 2 samples with support for all annotated introns	72.9%	2.5e-85
	TLR5	XP_010754180.1	Toll-like receptor 5	70.2%	4.6e-63
	TLR7	TLR7	Toll-like receptor 7	69.4%	3.8e-24
	IRAK1	EH28_12873	Interleukin-1 receptor-associated kinase 1-like	66.1%	2.8e-176
	TLR8	XP_010741343.1	Toll-like receptor 8	64.6%	5.5e-198
	IL-1β	IL-1β	Interleukin-1 beta-like	62.1%	9e-79
	TLR13	EH28_03283	Toll-like receptor 13; derived by automated computational analysis using gene prediction method: Gnomon. Supporting evidence includes similarity to: 48 proteins	49.7%	3.2e-82
	TNFRSF1B	TNFRSF1B	Tumor necrosis factor receptor superfamily member 1B; derived by automated computational analysis using gene prediction method: Gnomon. Supporting evidence includes similarity to: 4 proteins, and 100% coverage of the annotated genomic feature by RNAseq alignments, including 3 samples with support for all annotated introns	44.6%	1.2e-95
	IL-7	[STRING found no matching protein in its database]	-	-	-
	IL-18	[STRING found no matching protein in its database]	-	-	-
	IL-6	-	-	-	-
	IL-8	-	-	-	-
	TLR9	-	-	-	-
Anti-inflammatory proteins	AChE	ACHE	Acetylcholinesterase	91.3%	0.0
	CTSD	XP_010735640.1	Cathepsin D-like	90.4%	2e-213
	nAChRα7	EH28_10415	Neuronal acetylcholine receptor subunit alpha-7; derived by automated computational analysis using gene prediction method: Gnomon. Supporting evidence includes similarity to: 11 proteins	89.1%	6.5e-24
	NLRC3	EH28_01217	Protein NLRC3-like	88.2%	4e-57
	NLRX1	NLRX1	NLR family member X1	86.9%	0.0
	CTSS	CTSS	Cathepsin S	85.7%	3.5e-34
	CTSL	EH28_09355	Cathepsin L1-like	85.4%	1.8e-172
	BChE	XP_010750341.1	Acetylcholinesterase-like	76.8%	1.2e-267
	IL-10	IL10	Interleukin-10	76.4%	3.1e-34
	TGF-β	XP_010748216.1	Transforming growth factor beta-1-like	72.5%	8.8e-155
	NLRC5 Iso 1	NLRC5	NLR family CARD domain containing 5	70.3%	0.0
	NLRC5 Iso 2	NLRC5	NLR family CARD domain containing 5; protein NLRC5-like	59.9%	4e-170

Cell marker proteins of the gilthead seabream sequence showed percentages of identity with that of yellow croaker ranging between 67.1 and 91.4%. PHOX40 was the protein with the highest percentage (91.4%), followed by CSF1R (91%) and PHOX22 (89.7%). MHC-II showed the lowest percentage of identity (67.1%) (Table 3).

Pro-inflammatory proteins derived from the gilthead seabream protein sequence presented percentages of identity with that of yellow croaker ranging between 44.6 and 97.8% (Table 3). The protein sequence of the NF-κB family, including NF-κB2 (97.8%), RelA (82.3%), NF-κB1 (82%), C-Rel (81.3%), and RelB (81.1%), was the subgroup with the highest percentage of identity*,* while the TNR superfamily, including TNFRSFa (72.9%) and TNFRSF1b (44.6%), presented the lowest percentage of identity. The adaptor protein sequences (STAT3, 96.7%; TRAF6, 89.1%; IκBKG, 83.2; MYD88, 83%; IRAK1, 66.1%), the protein sequence of the TLR family (TLR2, 85%; TLR5, 70%; TLR7, 69.4%; TLR8, 64.6%; TLR13, 49.7%), and the sequence of cytokines (TNF-α, 84.7%; IL-1β, 62.1%) showed an intermediate identity percentage range. No associations were found between the IL-6, IL-7, IL-8, IL-18, and TLR9 proteins of the two species.

The identity of anti-inflammatory proteins was more homogeneous than that of pro-inflammatory proteins, with a range of identity between 59.9 and 91.3% (Table 3). The cholinergic protein sequences (AChE, 91.3%; nAChRα7, 89.1%; BChE, 76.8%) showed the highest percentages of identity*,* the cathepsin sequence (CTSD, 90.4%; CTSS; 85.7%; CTSL, 85.4%) presented intermediate ranges, and the anti-inflammatory cytokines (IL-10, 76.4%; TGF-β1, 72.5%) presented lower percentages of identity*.* On the other hand, the NOD-like receptor family sequences showed a wider range, with NLRC3 (88.2%) and NLRX1 (86.9%) exhibiting higher identity percentages than NLRC5 isoforms 1 (70.3%) and 2 (59.9%)*.*

### Functional protein association network analysis

**Fig.1:**
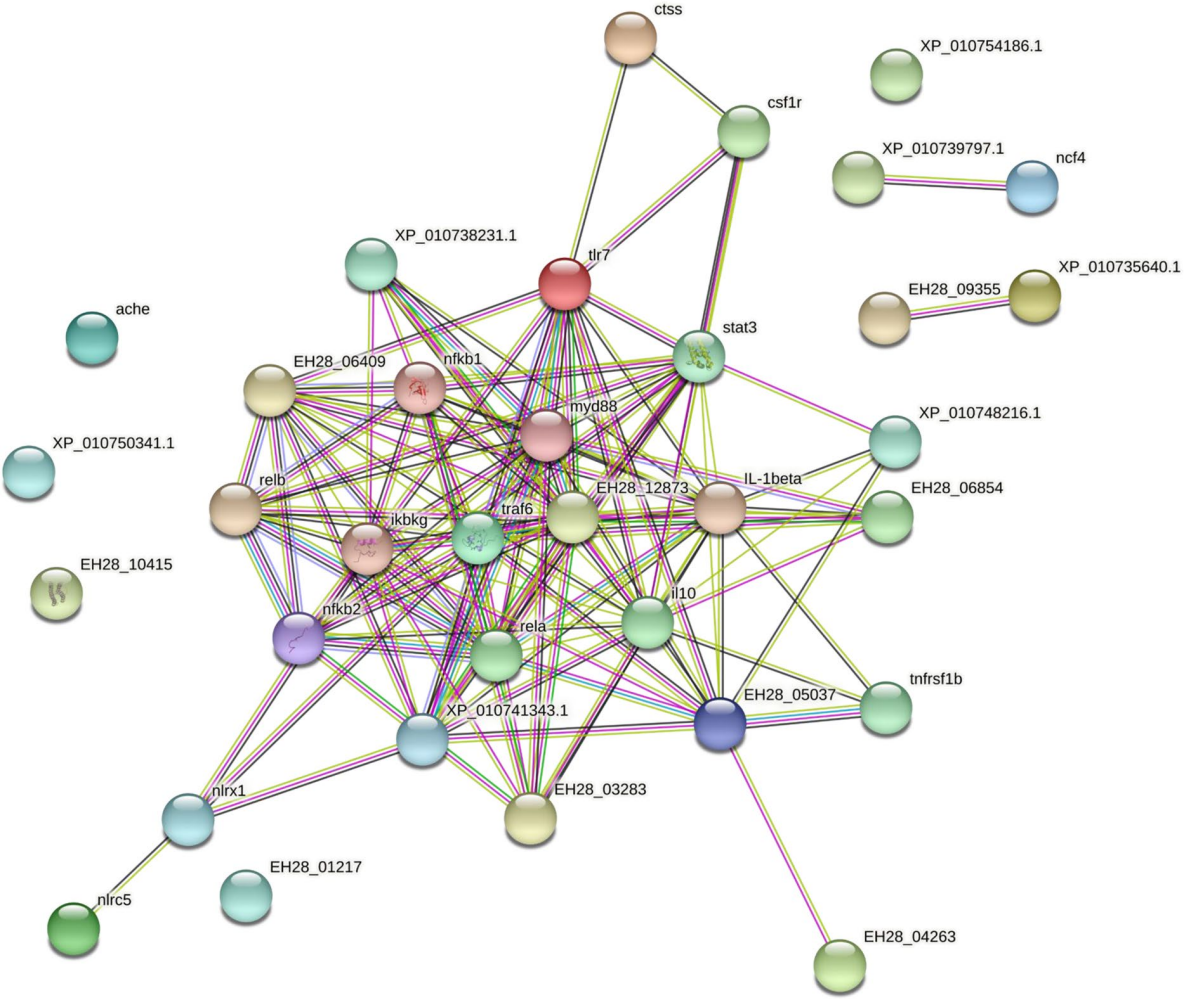
Representation of the node network (selected proteins) of *Larimichthys crocea* generated from the *Sparus aurata* protein sequence using the STRING database (see protein annotation in Table 3). The different edge colors indicate the type of evidence for each interaction: cyan = curated STRING database; purple = experimentally determined; green = gene neighborhood; blue = gene co-occurrence; light green = text mining; black = co-expression; lavender = protein homology (the types of evidence are not mutually exclusive)

The functional association network analysis revealed that 34 nodes (functional proteins) selected from the gilthead seabream sequence interacted in large yellow croaker (*Larimichthys crocea*) (Table 4). These 34 nodes represented 125 edges (specific and meaningful associations). The average node degree provided by STRING, which represents the number of interactions (at the score threshold) that a protein has on the average in the network, had a value of 7.35, while the clustering coefficient (a measure of how closely connected the nodes in the network are) had a value of 0.688 (Fig. 1).

**Table 4:** Functional protein association networks of *Larimichthys crocea* obtained from the gilthead seabream (*Sparus aurata*) protein sequence

Number of nodes	Number of edges	Average node degree	Avg. local clustering coefficient	Expected number of edges	PPI enrichment *p*-value
34	125	7.35	0.688	6	< 1.0e-16

### Gene expression analysis

Real-time PCR was used to analyze the expression profile of 4 cell markers, 24 pro-inflammatory genes, and 12 anti-inflammatory genes in fish skin samples collected 12 and 24 h after carrageenin or PBS injection.

#### Cell markers

**Fig. 2:**
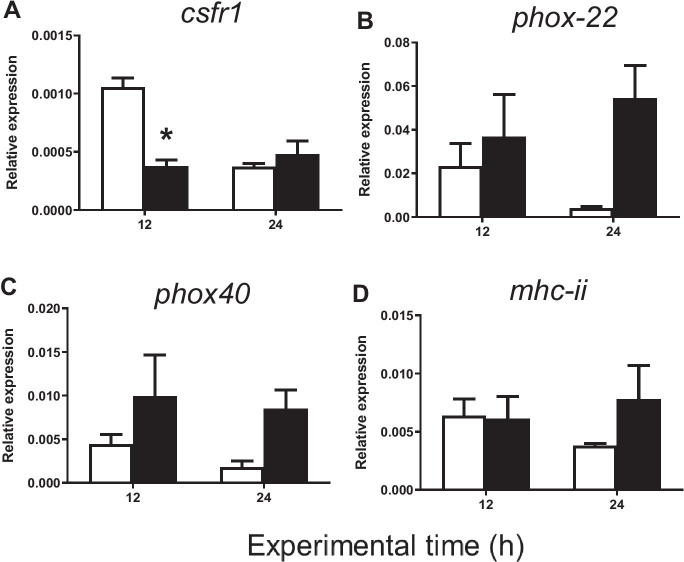
Relative expression of **A** *csfr1*, **B** *phox22*, **C** *phox40*, and **D** *mhciia* in gilthead seabream injected with PBS (control, white bars) and carrageenin (1%, black bars) analyzed in skin samples collected 12 and 24 h after injection. The bars represent the mean ± standard error of the mean (*n* = 4). The asterisks denote significant differences between the carrageenin and control groups (two-way ANOVA; *p* < 0.05)

The expression of the *csf1r* gene (macrophage marker) 12 h after carrageenin injection was significantly downregulated in comparison to that of control specimens (Fig. 2A). On the other hand, although no significant differences (*p* > 0.05) were observed either at 12 or at 24 h in the expression of *phox22* and *phox40* (acidophilic granulocyte markers) or *mhciia* (antigen-presenting cell marker) genes (Fig. 2B–2D), an ascendant tendency in the expression of *phox22* and *phox40* was found in the carrageenin group 24 h after injection with respect to the control group.

#### Pro-inflammatory genes

**Fig. 3:**
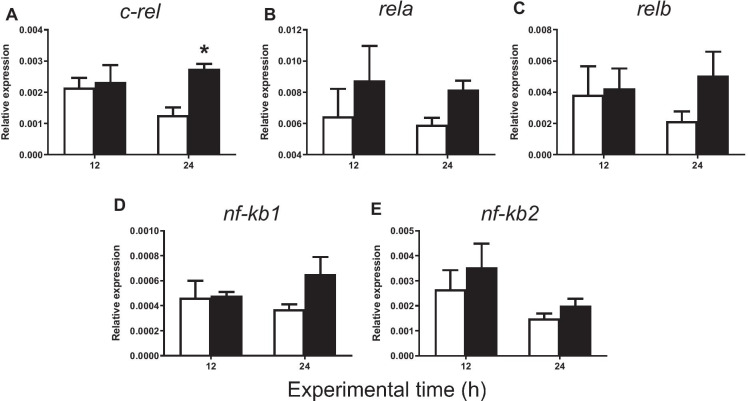
Relative expression of **A** *c-rel*, **B** *rela*, **C** *relb*, **D** *nf-κb1*, and **E** *nf-κb2* in gilthead seabream injected with PBS (control, white bars) and carrageenin (1%, black bars) analyzed in skin samples collected 12 and 24 h after injection. The bars represent the mean ± standard error of the mean (*n* = 4). The asterisks denote significant differences between the carrageenin and control groups (two-way ANOVA; *p* < 0.05)

**Fig. 4:**
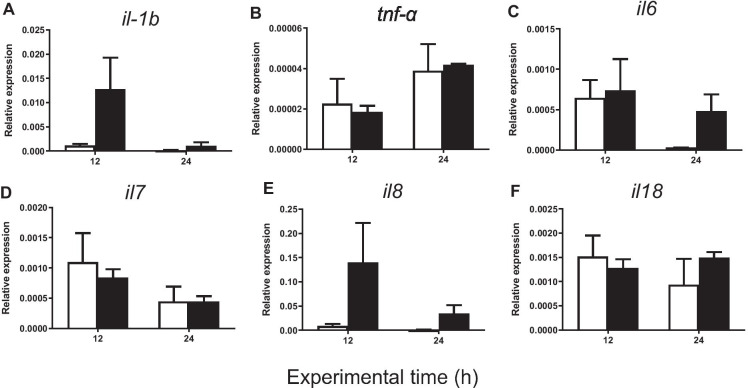
Relative expression of **A** *il-1β*, **B** *tnf-α*, **C** *il-6*, **D** *il-7*, **E** *il-8*, and **F** *il-18* in gilthead seabream injected with PBS (control, white bars) and carrageenin (1%, black bars) analyzed in skin samples collected 12 and 24 h after injection. The bars represent the mean ± standard error of the mean (*n* = 4). No significant differences were obtained neither between the carrageenin and control groups nor between the sampling time points (two-way ANOVA *p* < 0.05)

**Fig. 5:**
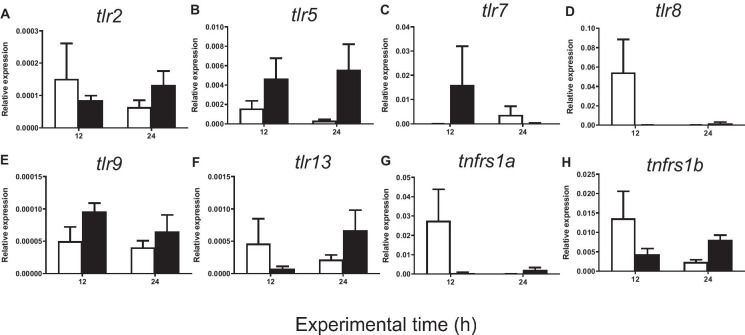
Relative expression of **A** *tlr2*, **B** *tlr5*, **C** *tlr7*, **D** *tlr8*, **E** *tlr9*, **F** *tlr13*, **G** *tnfrsf1a*, and **H** *tnfrsf1b* in gilthead seabream injected with PBS (control, white bars) and carrageenin (1%, black bars) analyzed in skin samples collected 12 and 24 h after injection. The bars represent the mean ± standard error of the mean (*n* = 4). No significant differences were obtained neither between the carrageenin and control groups nor between the sampling time points (two-way ANOVA; *p* < 0.05)

**Fig. 6:**
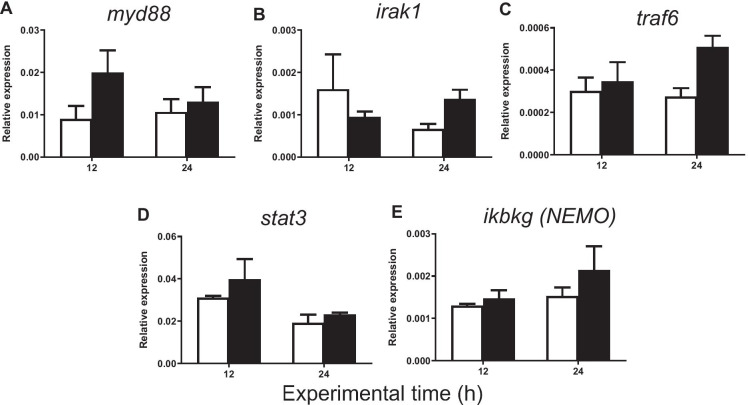
Relative expression of **A** *myd88*, **B** *irak1*, **C** *traf6*, **D** *stat3*, and **E** *ikbkg* (NEMO) in gilthead seabream injected with PBS (control, white bars) and carrageenin (1%, black bars) analyzed in skin samples collected 12 and 24 h after injection. The bars represent the mean ± standard error of the mean (*n* = 4). No significant differences were obtained neither between the carrageenin and control groups nor between the sampling time points (two-way ANOVA; *p* < 0.05)

The administration of carrageenin produced no statistically significant differences in the expression of c-*rel* between the carrageenin and control groups 12 h after injection. However, this gene was significantly upregulated at 24 h in the carrageenin group in comparison with the control group (Fig. 3A). Interestingly, no significant differences were found between the two groups in the gene expression of many other pro-inflammatory genes (*rela*, *relb*, *nf-κb1*, *nf-κb2*, *il-1β*, *tnf-α*, *il-6*, *il-7*, *il-8*, *il-18*, *tlr2*, *tlr5*, *tlr7*, *tlr8*, *tlr9*, *tlr13, tnfrsf1a*, *tnfrsf1b*, *myd88*, *irak1*, *traf6, stat3*, *ikbkg*) either at 12 or at 24 h after injection (Figs. 3, 4, 5 and 6).

#### Anti-inflammatory genes

**Fig. 7:**
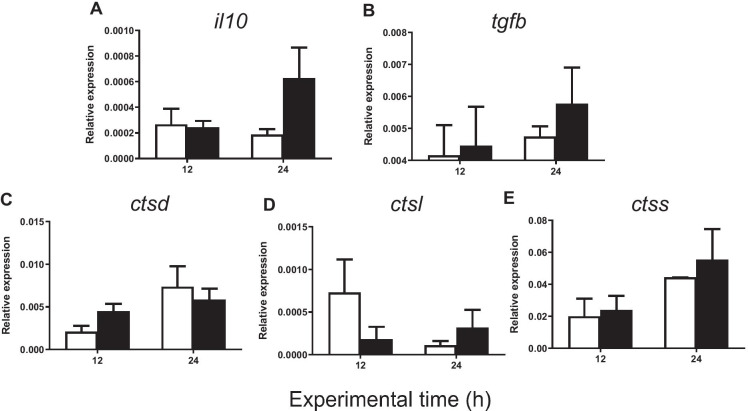
Relative expression of **A** *il10*, **B** *tgf-b*, **C** *ctsd*, **D** *ctsl*, and **E** *ctss* in gilthead seabream injected with PBS (control, white bars) and carrageenin (1%, black bars) analyzed in skin samples collected 12 and 24 h after injection. The bars represent the mean ± standard error of the mean (*n* = 4). No significant differences were obtained neither between the carrageenin and control groups nor between the sampling time points (two-way ANOVA; *p* < 0.05)

**Fig. 8:**
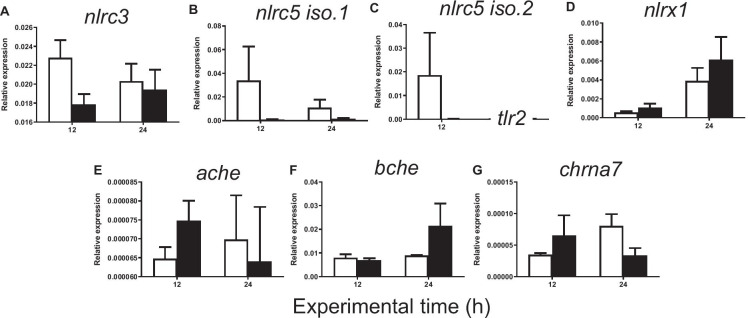
Relative expression of **A** *nlrc3*, **B** *isoform 1 of nlrc5*, **C** *isoform 2 of nlrc5*, **D** *nlrx1*, **E** *ache*, **F** *bche*, and **G** *chrna7* in gilthead seabream injected with PBS (control, white bars) and carrageenin (1%, black bars) analyzed in skin samples collected 12 and 24 h after injection. The bars represent the mean ± standard error of the mean (*n* = 4). No significant differences were obtained neither between the carrageenin and control groups nor between the sampling time points (two-way ANOVA; *p* < 0.05)

**Fig. 9:**
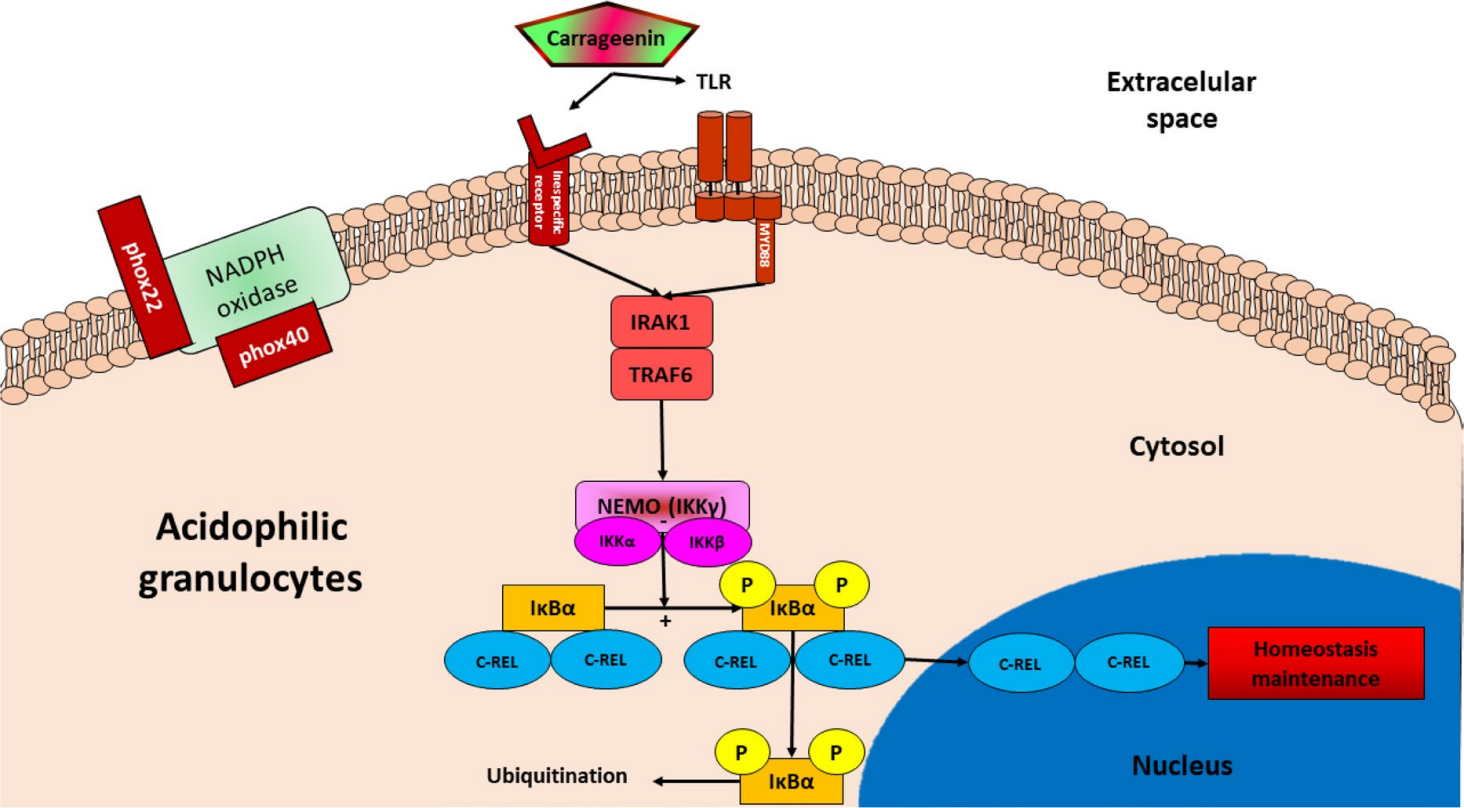
Proposed schematic inflammatory model of carrageenin–transduction pathway for acidophilic granulocytes in the skin of gilthead seabream. The + and − symbols represent induction and repression interactions, respectively

The injection of carrageenin resulted in no significant changes in the expression of anti-inflammatory genes (*il-10*, *tgf-β1*, *ctsd*, *ctsl*, *ctss*, *nlrc3*, *nlrc5* isoforms 1 and 2, *nlrx1*, *ache*, *bche*, and *chrna7*) with respect to the control group either at 12 or at 24 h (Figs. 7 and 8). All the results of the gene expression analysis are summarized in a schematic inflammatory model shown in Fig. 9.

## Discussion

Although inflammation has been extensively studied in mammals, it is possible that not all the transduction pathways of this process have been conserved in the evolution of vertebrates (Byadgi et al. 2018; Savan and Sakai 2006). To our knowledge, this is the first study to investigate the conservation of inflammatory pathways in fish using the STRING database to identify functional associations of inflammation-related proteins applicable to the gilthead seabream (Balasch and Tort 2019; Cordero et al. 2017; Sarropoulou et al. 2007; Silva-Marrero et al. 2017; Tsakogiannis et al. 2019). In our analysis, *L. crocea*, a species of particular interest for the marine aquaculture of the north-western Pacific, was the species whose protein sequence seemed to most closely match that of the gilthead seabream (Tan et al. 2019; Wang et al. 2016). It can be assumed that the analyzed proteins are highly conserved between the gilthead seabream and yellow croaker, since protein sequences with > 25% identity are considered orthologs (genes homologous by speciation) at the protein level and could be used to predict their molecular functions (Konaté et al. 2017). Furthermore, none of the studied sequences showed a percentage of identity less than 30%, the threshold below which protein structures degenerate (Gilson et al. 2017). Moreover, the 34 nodes obtained represented 125 edges, while the number of expected edges for a random set of proteins of the yellow croaker genome was 4. This high number suggests that the selected proteins are, at least partially, biologically connected (not necessarily physically bound but with some specific and meaningful associations), suggesting a shared function. These proteins are quite likely associated with inflammation (Postlethwait et al. 2000). It is noteworthy that the level of molecular conservation of cell markers CSF1R (also known as macrophage colony-stimulating factor receptor, MCSFR) and PHOX40, and PHOX22 between the gilthead seabream and large yellow croaker was significantly high (identity percentage close to 90%). These are important biomarkers for macrophages and acidophilic granulocytes (Grayfer et al. 2009; Meseguer et al. 1994). Interestingly, although the catalytic tyrosine kinase domains of CSF1R are highly conserved among vertebrates, it seems that *csf1r* genes exhibit a low sequence identity (especially in their extracellular domains), which could be reflected in the low conservation of the CSF1R ligand and the distinct macrophage functionality across divergent species (Grayfer et al. 2018). Likewise, the catalytic domains of PHOX22 and PHOX40, which are transmembrane and cytosol proteins, respectively, of the NADPH oxidase enzyme system of gilthead seabream acidophilic granulocytes, are highly conserved (Belambri et al. 2018; Kawahara and Lambeth 2007; Meseguer et al. 1994; Sepulcre et al. 2002). In contrast, the basic pattern of MHC variation in mammals (Dijkstra et al. 2013; Yamaguchi and Dijkstra 2019) that has remained in fish with a high level of allelic/haplotype diversification explains the lower percentage of identity of MHC-II compared to the other cell markers in our study.

Regarding pro-inflammatory proteins, we found a high conservation level of proteins related to the NF-κB family, consisting of five polypeptide subunits-RelA (p65), RelB, C-Rel, NF-κB1 (p50/p105), and NF-κB2 (p52/p100). This could be due to the key role that this transcription factor plays in the activation and release of pro-inflammatory cytokines, as well as its implication in the expression of pro-survival molecules involved in the regulation of important processes, such as the immune response and homeostasis maintenance (Etemadi et al. 2015; Gugasyan et al. 2004; Mulero et al. 2019; Napetschnig and Wu 2013). This is also supported by the relatively highly conserved adaptors and regulatory proteins (STAT3, TRAF6, IκBKG, MYD88, and IRAK1), which might be critical for maintaining a minimal threshold of NF-κB signalling and whose activity could also be regulated by intracellular homeostatic processes, such as oxidative stress, metabolite changes, and DNA damage (Hinz et al. 2010; Linares et al. 2013; Matsuzawa et al. 2005; Moscat and Diaz-Meco 2016; Rezaeian et al. 2017; Tang et al. 2013; Tzeng et al. 2013; Zhang et al. 2016).

Likewise, the high percentage of identity (around 85%) of anti-inflammatory proteins, such as cholinergic proteins and cathepsins, could also be related to their regulatory function. For instance, facing an inflammatory response, the nervous system of higher organisms can release acetylcholine through the efferent vagus nerve, which is activated by AChE and BChE, and inhibit the production of pro-inflammatory cytokines in macrophages via activation of the α7-nicotinic homopentameric receptor (α7nAChR) (Zila et al. 2017). It has been observed in human cell lines that NOD-like receptors, such as NLRC3, may also downregulate the expression of NF-κB transcription factor by TRAF6 ubiquitination, while mitochondrial NLRX1 and NLRC5 limit the NF-κB activation by interfering with the interactions of the TRAF6-IKK (Iκ-B kinase) protein complex and the IKKα protein, respectively (Cui et al. 2010; Schneider et al. 2012; Xia et al. 2011). Cathepsins, for their part, not only are lysosomal endonucleases that negatively modulate inflammation but are also involved in cell survival and apoptosis, participating, among other functions, in neutrophil recruitment (CTSL), caspase activation (CTSD), and process and maturation of the associated invariant chain of MHC-II (CTSS) (Conus et al. 2008; Conus and Simon 2008; Li et al. 2015).

It is important to emphasize the ancient genome duplication that occurred in teleosts, whereby an estimated 30% of genes were duplicated. This could explain the main protein sequence differences found in our assay (Postlethwait et al. 2000). It could also support the high conservation of molecules involved in the regulatory pathways of the immune response (such as the NF-κB family transcription factors). On the other hand, secondary mediators (such as pro-inflammatory and anti-inflammatory cytokines and proteins of the TLR and NOD families and the TNR superfamily) could vary structurally or sequentially depending on the organism, preserving their functions to a greater or lesser extent in most teleost fishes as paralogues (multiple copies) (Mulero et al. 2019; Secombes et al. 2011; Wang and Secombes 2013). This also explains the presence of two isoforms of NLRC5 in the gilthead seabream, whose protein sequence seems to coincide to some degree with the NLRC5 protein sequence of *L. crocea*, suggesting that NLRC5 isoform 1 of the gilthead seabream could be more conserved than isoform 2.

To validate the in silico results obtained from the STRING database and to confirm the conservation of the molecules involved in the inflammatory response of the gilthead seabream, an in vivo analysis of the gene expression was performed using carrageenin as a stimulus of inflammation according to the schema shown in Fig. 9. The concentration of injected carrageenin (50 µl of 1% carrageenin) and the sampling times (12 and 24 h after injection) were carefully chosen considering the available data from studies on mammals (Levy 1969; Morris 2003; Winter et al. 1962). Nonetheless, although proteins seem to be relatively highly conserved, some key considerations are important for understanding the functional differences between studied organisms and the effects of carrageenin on the gilthead seabream. For instance, unlike in mammals, leucocytes in fish should migrate from the head kidney (the main hematopoietic organ) to the injured site when an insult is perceived by local cells (Meseguer et al. 1995), such as the presence of carrageenin in this study. Therefore, infiltrating acidophilic granulocytes of the gilthead seabream, which are functionally equivalent to mammalian neutrophils (Meseguer et al. 1994; Sepulcre et al. 2002), should be the first cells to be recruited to the inflammation site, followed by macrophages (Kolaczkowska and Kubes 2013; Medzhitov 2008; Nathan 2006; Nguyen-Chi et al. 2015).

The observed downregulation of *csf1r* expression 12 h after carrageenin injection suggests a cytotoxic effect on macrophages since *csf1r* is an important biomarker for teleost fish macrophage survival, proliferation, and differentiation (Hanington et al. 2009; Roca et al. 2006). Studies on mice (*Mus musculus*), guinea pigs (*Cavia porcellus*), and zebrafish (*Danio rerio*) have shown that carrageenin can specifically deplete macrophages without affecting neutrophil activity (Kaneda et al. 1991; Mitsuyama et al. 1982; Phelps and Neely 2007), which lends weight to the hypothesis of a cytotoxic effect of carrageenin on gilthead seabream macrophages. Furthermore, the observed increase tendency of *phox22* and *phox40* at both 12 and 24 h after carrageenin injection suggests the activation or increased recruitment of acidophilic granulocytes to the inflammation site at that time. However, further studies are needed to elucidate the effect of carrageenin at the subcellular level and confirm these hypotheses (Belambri et al. 2018).

Otherwise, and regarding the subunits of NF-κB transcription factor in mammals, it would be important to consider that RelA:NF-κB1 heterodimer is the principal product that induces the canonical signalling pathway of inflammation. RelB:NF-κB2 constitutes the non-canonical or alternative activation pathway, leaving c-REL subunit as a secondary molecule in this process (Mulero et al. 2019). Interestingly, the upregulated expression of c-*rel* at 24 h found in our study after carrageenin injection could suggest an alternative pathway of activating inflammation in fish. Thus, c-REL homodimers could combinate activating the transcription of inflammation-related genes, since c-REL heterodimers with other NF-κB components have not been documented (Mulero et al. 2019). In addition, the trigger of a faster inflammatory response than in mammals could also explain that the expression of cytokine genes was not significantly altered in the time tested. Taken together, these results suggest that the activation of these genes might be the first step in the termination of the innate immune response in order to restore homeostasis (Gugasyan et al. 2004).

## Conclusion

These results indicate that the molecules involved in the inflammatory process, especially the proteins involved in the regulation of the inflammatory response, are mostly conserved among fish species, but their main functions can vary depending on the species due to the different orthologues present in each. Furthermore, the inflammation induced by subcutaneous carrageenin injection (at least at the tested dose) in the gilthead seabream abates within hours. The mechanisms necessary to terminate the inflammation response and recover skin homeostasis are activated between 12 and 24 h after injection. The in silico and gene expression analyses performed in this study might contribute to the identification of the main mechanisms of the acute inflammatory response. Our results have implications not only for applications in the aquaculture sector but also for basic research. Future studies using this characteristic pro-inflammatory mucopolysaccharide are needed to elucidate the complex mechanism of inflammation related to diseases or dietary supplementation in fish.

## Data Availability

The datasets generated during and/or analyzed during the current study are available from the corresponding author on reasonable request.
